# Transcription Pattern of Neurotrophic Factors and Their Receptors in Adult Zebrafish Spinal Cord

**DOI:** 10.3390/ijms241310953

**Published:** 2023-06-30

**Authors:** Pietro Cacialli, Serena Ricci, Maurizio Lazzari, Liliana Milani, Valeria Franceschini

**Affiliations:** Department of Biological, Geological and Environmental Sciences, University of Bologna, 40126 Bologna, Italy; serena.ricci@outlook.it (S.R.); maurizio.lazzari@unibo.it (M.L.); liliana.milani@unibo.it (L.M.); valeria.franceschini@unibo.it (V.F.)

**Keywords:** zebrafish, neurotrophin, neurotrophin-receptor TRK, spinal cord, aquatic animal model

## Abstract

In vertebrates, neurotrophins and their receptors play a fundamental role in the central and peripheral nervous systems. Several studies reported that each neurotrophin/receptor signalling pathway can perform various functions during axon development, neuronal growth, and plasticity. Previous investigations in some fish species have identified neurotrophins and their receptors in the spinal cord under physiological conditions and after injuries, highlighting their potential role during regeneration. In our study, for the first time, we used an excellent animal model, the zebrafish (*Danio rerio*), to compare the mRNA localization patterns of neurotrophins and receptors in the spinal cord. We quantified the levels of mRNA using qPCR, and identified the transcription pattern of each neurotrophin/receptor pathway via in situ hybridization. Our data show that *ngf/trka* are the most transcribed members in the adult zebrafish spinal cord.

## 1. Introduction

Neurotrophins and their receptors modulate different events in the central and peripheral nervous systems, such as neuronal survival, differentiation, plasticity, and axonal growth. Neurotrophic factors and receptors have been found in different vertebrates. Nerve growth factor (NGF) was the first member that was identified in detail [1,2,3]. NT4 was first isolated from *Xenopus laevis* [4], and NT5 was first isolated from the rat [5,6]. In fish species, previous studies showed that from two neurotrophin gene ancestors, a couple of paralogues, BDNF–NT4/5 and NGF–NT3, originated for duplication following the split of jawless fish, but before the split of cartilaginous fish from the common vertebrate lineage [7]. In parallel to this genome duplication event, in Osteichtyes (teleosts), also known as bony fishes, NGF and NT 6/7 originated from a further duplication of an ancestral gene not identified in other vertebrates [8,9,10,11]. Previous phylogenetic studies clearly show that NT4 and NT5 are orthologues [12]. NT6 and NT7 have been suggested to be paralogues [13], probably resulting from the duplication of an ancestral NGF fish gene. Concerning the receptors, they appear to be conserved in vertebrates, including mammals, with the sole exception of lineage-specific duplications in teleost fishes [14], resulting in the presence of five tyrosine protein kinase receptors *Trka*; *Trkb*; *Trkb2*; *Trkc1*; and *Trkc2* [15,16].

As we mentioned above, neurotrophic factors and their receptors play several important roles in the brain and especially the spinal cord of different vertebrates [17,18]. In the spinal cord, neurotrophins and receptors can modulate repair after a traumatic event [19,20]. Indeed, recent functional in vivo studies in mouse and rat models showed that cell transplants [21], including engineered stem cells (that overexpress neurotrophins and/or receptors), can affect the function of spinal neurons and circuits [22]. However, further studies using emerging animal models, such as the zebrafish (*Danio rerio*), could be useful to elucidate the specific mechanisms of this damage. The use of the zebrafish model in scientific research leads many advantages, such as low costs, reduced use of spaces, and high fecundity. The anatomy of different organs and tissues is conserved during vertebrate evolution; indeed, the zebrafish has become a popular animal model for several fields of research: development biology, genetics, hematology, neuroscience, cardiology, etc. [23]. The regenerative properties of the zebrafish (organs and tissues) are a constantly fascinating subject of study for the scientific community to identify new molecular pathways that could represent a potential alternative to understand how organs can be restored when tissues and cells are damaged by diseases or traumatic events [24]. The zebrafish is an excellent model to study the anatomy as well as molecular and cellular biology of the vertebrate spinal cord [25,26]. In detail, this model is widely used to study the role of specific pathways in the spinal cord under physiological conditions and after injuries [27,28,29]. The basic structure of the spinal cord in zebrafish (as in other fish) resembles that of other vertebrates [30,31]. It runs dorsal and lengthwise to the fish body in the neural canal of the vertebral column. In the transverse section of spinal cord, two regions are clearly distinct, central and peripheral. In adult zebrafish, neurotrophins and their receptors were extensively described in different tissues and organs: the brain, kidney, ovary, testis, and the sensory and olfactory organs [32,33,34,35,36,37,38,39,40,41]. However, there are no extensive studies regarding the specific identification and quantification of the mRNA localization pattern of neurotrophins and their receptors in the adult zebrafish spinal cord. Therefore, the aim of the present study is to gain insights into the anatomical localization of neurotrophin and its Trk receptor transcripts in the spinal cord of the zebrafish, in order to inspire further experimental investigations concerning spinal cord regeneration.

## 2. Results

### 2.1. Experimental Design Outline for qPCR

**Figure 1:**
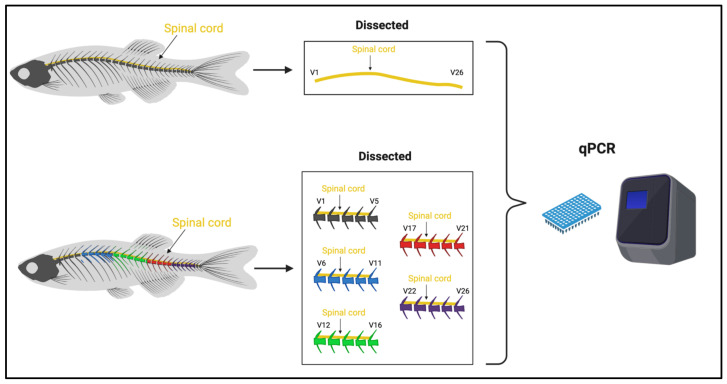
Schematic experimental design outline for qPCR. Whole adult zebrafish spinal cord and five segments of adult zebrafish spinal cord were dissected and used for RNA extraction and qPCR.

For qPCR experiments, we first measured neurotrophin and receptor transcription levels in the whole spinal cord of adult zebrafish (comparing with the whole brain as the control). Next, we measured neurotrophin and receptor transcription levels by dividing the spinal cord into five segments (from the first to the last vertebra), to verify if variations in transcription levels were present in a specific region (Figure 1).

### 2.2. Quantitative Analysis of Neurotrophin and Receptor Transcription Levels in Adult Zebrafish Spinal Cord

**Figure 2:**
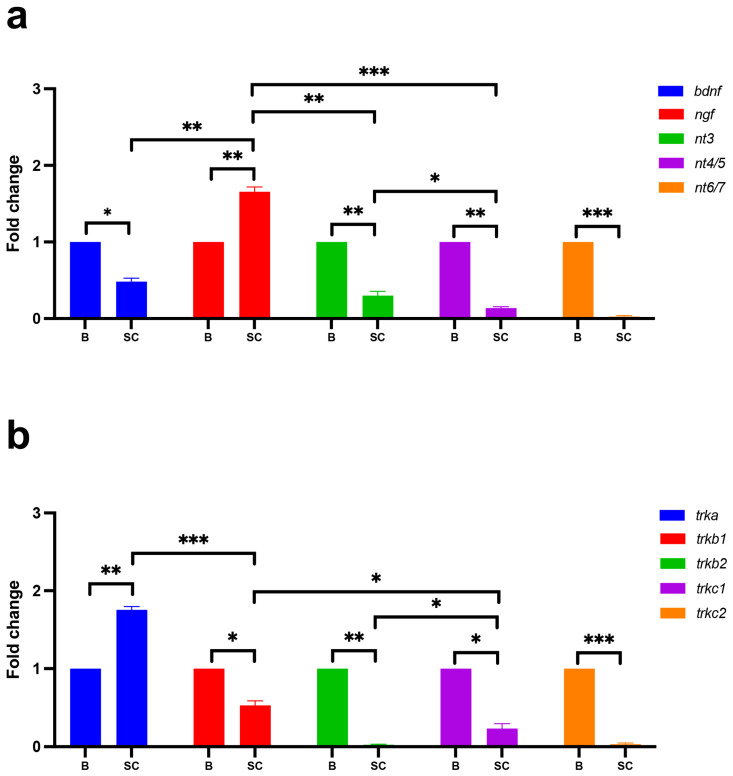
qPCR analysis of neurotrophins and receptors in the whole adult zebrafish spinal cord (SC) compared with the whole brain (B). (**a**) *Ngf* is the most transcribed member of neurotrophins, followed by *bdnf* and *nt3*, more transcribed than *nt4/5*. *Nt6/7* is undetectable. Statistical analysis was completed using an unpaired *t*-test (*n* = 5 animals used). * *p* < 0.01; ** *p* < 0.001; *** *p* < 0.0001. (**b**) qPCR analysis for five receptors of tyrosine kinase: *trkA*; *trkb1*; *trkb2*; *trkc1*; and *trkc2*. The receptor *trka* is the most transcribed receptor compared with *trkb1* and *trkc1*. *Trkb2* and *trkc2* are undetectable. Statistical analysis was completed using an unpaired *t*-test, (n = 5 animals used). * *p* < 0.01; ** *p* < 0.001; *** *p* < 0.0001.

To measure the transcription level of each gene for neurotrophins and their specific receptors in the adult zebrafish spinal cord we used a qPCR experimental approach. First, we analyzed the transcription levels of neurotrophic factors and receptors in the whole spinal cord (SC) and compared these with whole brain tissue (B) as the control. We found that *bdnf* mRNA presents less expression in the spinal cord compared with the whole brain. *Ngf* mRNA is highly expressed in the spinal cord compared with the brain, and it is the most expressed transcript in the whole adult zebrafish spinal cord, compared to other neurotrophic factors. *Nt4/5* presented a low level of transcription in the spinal cord, and *nt6/7* was undetected in this tissue (Figure 2a). Concerning the five receptors, we found that *trka* mRNA is enriched in the spinal cord compared with brain tissue, and it is the most transcribed. *Trkb1* and *trkc1* present less expression in the spinal cord compared with the brain. *Trkb2* and *trkc2* were undetected in the spinal cord (Figure 2b). Next, as we mentioned above, we analyzed the neurotrophin and receptor transcription levels in five sequential segments of the spinal cord, and we did not find many differences, except for a slight variation of *bdnf* in the segments V1–5 (lower transcription level in this region) (Supplementary Figure S1a,b). Our results confirmed that *ngf* and its receptor *trka* are the most transcribed members in the adult zebrafish spinal cord.

### 2.3. Differential Transcription Pattern of Neurotrophins in Adult Zebrafish Spinal Cord

**Figure 3:**
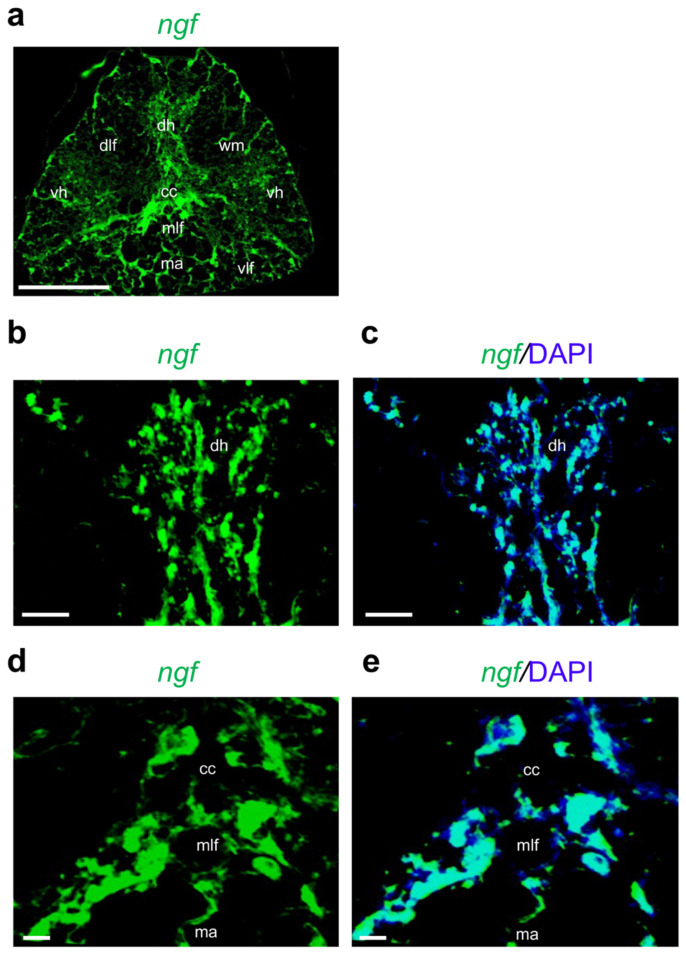
Fluorescence in situ hybridization for *ngf* in adult zebrafish spinal cord. (**a**) *Ngf* mRNA distribution in adult zebrafish spinal cord (low magnification of transversal section). (**b**) *Ngf* mRNA in dorsal horn (dh) region (high magnification). (**c**) *Ngf* mRNA in dorsal horn (dh) region, and cell nuclei are labeled with DAPI (blue). (**d**) *Ngf* mRNA distributed around central canal (cc); in medial longitudinal fasciculi (mlf) and Mauthner axons (ma). (**e**) *Ngf* mRNA distributed around central canal (cc); in medial longitudinal fasciculi (mlf) and Mauthner axons (ma), and cell nuclei are labeled with DAPI (blue). Scale bars: 100 μm (**a**); 50 μm (**b**–**e**).

In order to identify the transcription pattern of neurotrophins except *nt6/7* (undetected) in adult zebrafish spinal cord, we performed fluorescence in situ hybridization on transversal paraffin sections. We chose the region V17–21, since it is widely used to identify morphological features of the adult spinal cord in regenerative studies in zebrafish [27,42]. We found that the *ngf* transcript is the most coherently transcribed compared to other neurotrophins, using qPCR analysis (Figure 3a). Based on the morphological features described by Stil and Drapeau [43], we identified several structures: dorsal horn (dh); ventral horns (vh); white matter (wm); central canal (cc); Mauthner axons (ma); dorsal, ventral, and medial longitudinal fasciculi (dlf, vlf, and mlf, respectively).

**Figure 4:**
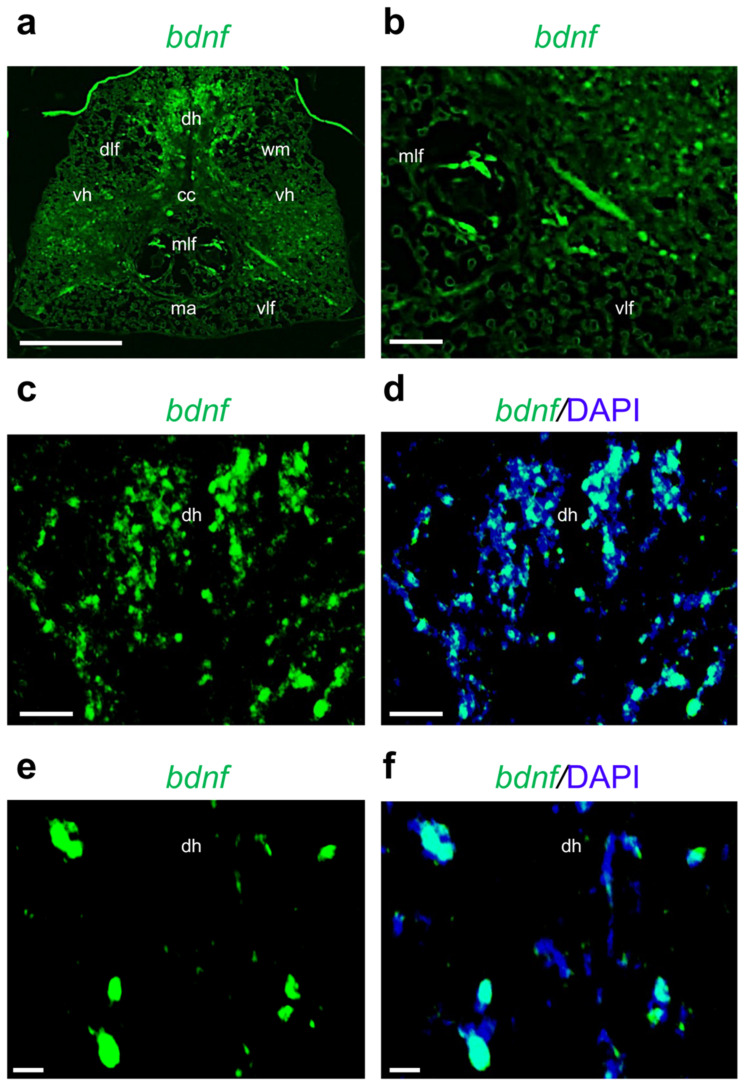
Fluorescence in situ hybridization for *bdnf* in adult zebrafish spinal cord. (**a**) *Bdnf* mRNA distribution in adult zebrafish spinal cord (low magnification of transversal section). (**b**) *Bdnf* mRNA in medial longitudinal fasciculi (mlf), high magnification. (**c**) *Bdnf* mRNA in dorsal horn (dh) region (high magnification). (**d**) *Bdnf* mRNA in dorsal horn (dh) region, and cell nuclei are labeled with DAPI (blue). (**e**) *Bdnf* mRNA localization in cells by high magnification. (**f**) *Bdnf* mRNA localization in cells by high magnification, and cell nuclei are labeled with DAPI (blue). Scale bars: 100 μm (**a**); 50 μm (**b**–**d**); 25 μm (**e**,**f**).

In detail, *ngf* mRNA is predominantly localized in cells around the central canal, in the dorsal and ventral horns, white matter, and Mauthner axons (Figure 3b–e). *Bdnf* is mainly transcribed in the medial longitudinal fasciculi (Figure 4a,b) and dorsal horn (Figure 4c,d). We can appreciate the morphology of *bdnf*-transcribing cells using high magnification (Figure 4e,f).

**Figure 5:**
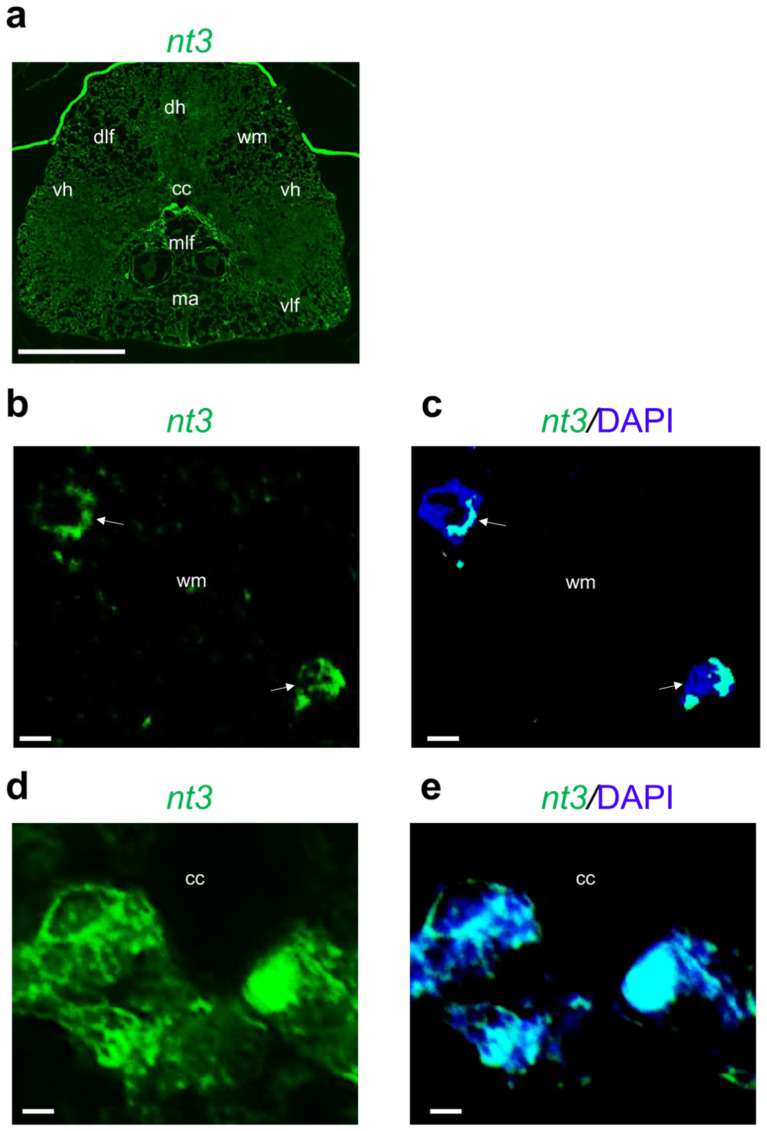
Fluorescence in situ hybridization for *nt3* in adult zebrafish spinal cord. (**a**) *Nt3* mRNA distribution in adult zebrafish spinal cord (low magnification of transversal section). (**b**) *Nt3* mRNA in white matter (wm) region (high magnification) white arrow indicate the expressing cells. (**c**) *Nt3* mRNA in white matter (wm) region, and cell nuclei are labeled with DAPI (blue). white arrow indicate the expressing cells (**d**) *Nt3* mRNA distributed in cells around central canal (cc); (**e**) *Nt3* mRNA distributed around central canal (cc), and cell nuclei are labeled with DAPI (blue). Scale bars: 100 μm (**a**); 25 μm (**b**–**e**).

Interestingly, *nt3* mRNA is specifically transcribed in cells around the central canal and white matter (Figure 5a–e).

**Figure 6:**
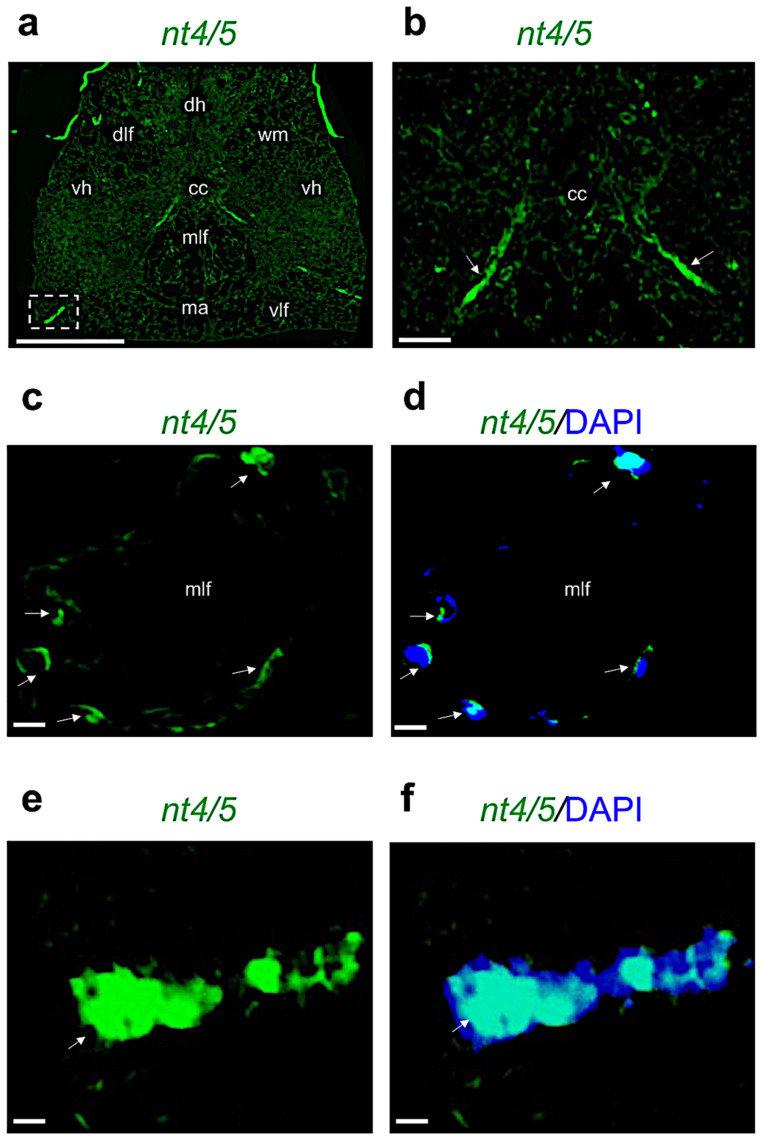
Fluorescence in situ hybridization for *nt4/5* in adult zebrafish spinal cord. (**a**) *Nt4/5* mRNA distribution in adult zebrafish spinal cord (low magnification of transversal section). (**b**) *Nt4/5* mRNA around central canal region (high magnification). (**c**) *Nt4/5* mRNA in medial longitudinal fasciculi (mlf) region. (**d**) *Nt4/5* mRNA in medial longitudinal fasciculi (mlf) region, and cell nuclei are labeled with DAPI (blue). (**e**) *Nt4/5* mRNA-transcribing cells in the basal part of ipsilateral bundles, high magnification of the region in the white rectangle (relative to (**a**)). (**f**) *Nt4/5* mRNA-transcribing cells in the basal part of ipsilateral bundles and DAPI. White arrow in (**c**–**f**) indicate *nt4* expressing cells. Scale bars: 100 μm (**a**); 50 μm (**b**); 25 μm (**c**–**f**).

In contrast, *nt4/5* mRNA is transcribed starting close to the central canal, sending ipsilateral projection to the ventro–lateral edge of the spinal cord (Figure 6a,b), and in a few cells of the medial longitudinal fasciculi (Figure 6c,d). We can appreciate *nt4/5* mRNA-transcribing cells in the basal part of ipsilateral bundles (Figure 6e,f).

### 2.4. Distribution of Neurotrophin Receptor Tyrosine Kinase mRNAs in Adult Zebrafish Spinal Cord

**Figure 7:**
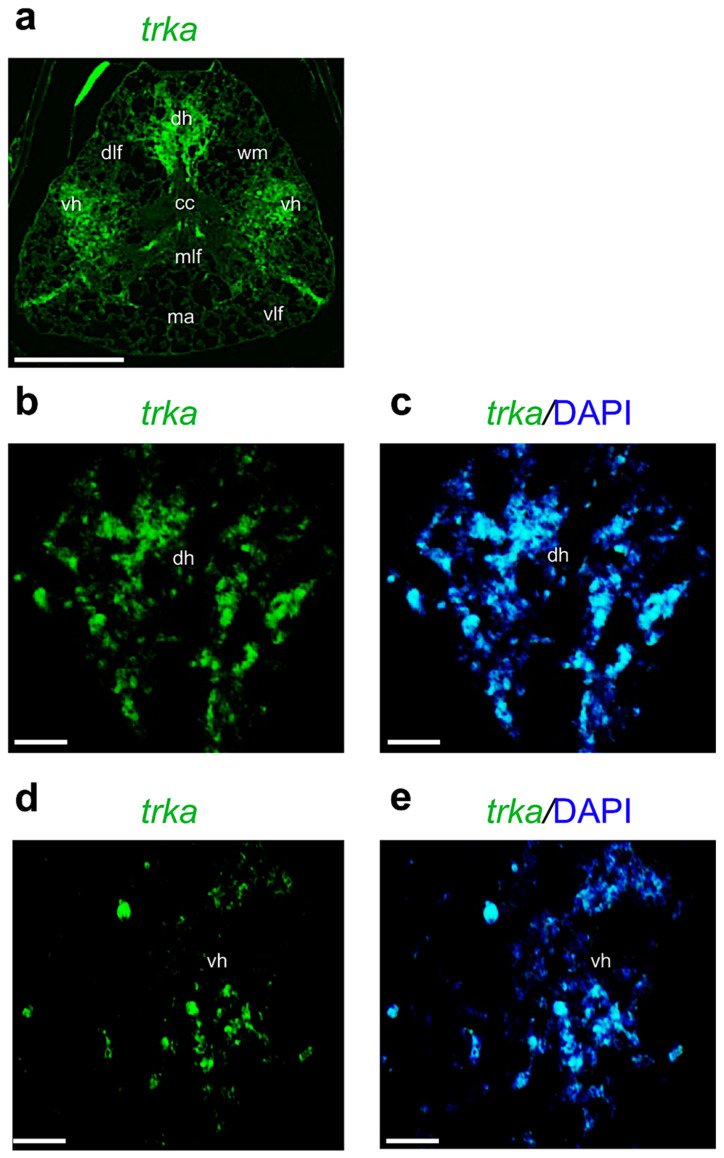
Fluorescence in situ hybridization for *trka* in adult zebrafish spinal cord. (**a**) *Trka* mRNA distribution in adult zebrafish spinal cord (low magnification of transversal section). (**b**) *Trka* mRNA in dorsal horn (dh) region (high magnification). (**c**) *Trka* mRNA in dorsal horn (dh) region, and cell nuclei are labeled with DAPI (blue). (**d**) *Trka* mRNA distributed in ventral horn (vh). (**e**) *Trka* mRNA distributed in ventral horn and cell nuclei are labeled with DAPI (blue). Scale bars: 100 μm (**a**); 50 μm (**b**–**e**).

Concerning the receptors, we found that *trka* mRNA is highly transcribed in the adult zebrafish spinal cord; this result confirms the data we obtained with qPCR. *Trka* mRNA is transcribed in the dorsal and ventral horns, and in cells around the central canal (Figure 7a–e).

**Figure 8:**
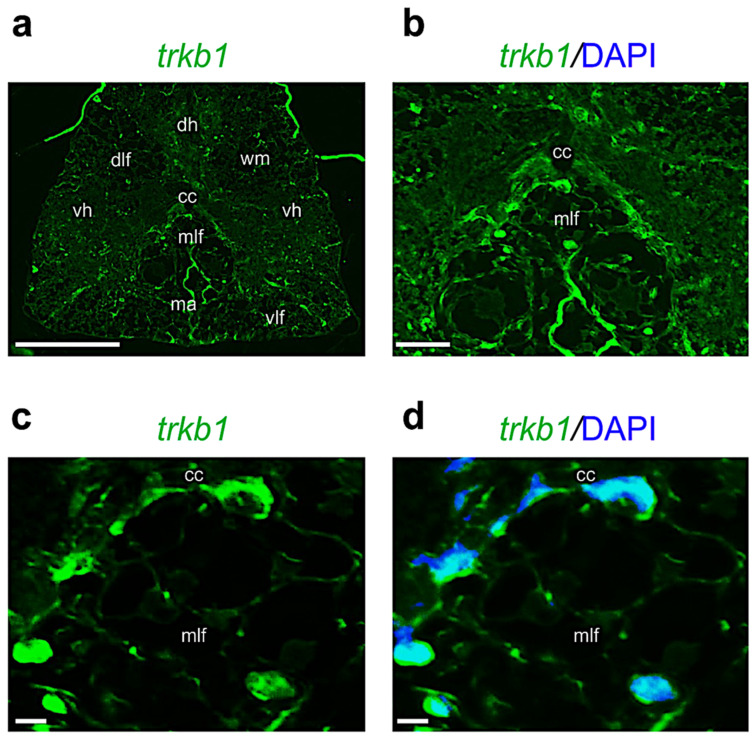
Fluorescence in situ hybridization for *trkb1* in adult zebrafish spinal cord. (**a**) *Trkb1* mRNA distribution in adult zebrafish spinal cord (low magnification of transversal section). (**b**) *Trkb1* mRNA transcribed in cells around central canal (cc) and medial longitudinal fasciculi regions (mlf). (**c**) High magnification of *trkb1* mRNA-transcribed cells around central canal (cc) and medial longitudinal fasciculi regions (mlf). (**d**) High magnification of *trkb1* mRNA-transcribing cells, and cell nuclei are labeled with DAPI (blue) around central canal (cc) and medial longitudinal fasciculi regions (mlf). Scale bars: 100 μm (**a**); 50 μm (**b**); 25 μm (**c**,**d**).

*Trkb1* mRNA is mainly transcribed in cells around the central canal and medial longitudinal fasciculi; a low transcription level is present in a few cells of the dorsal horn (Figure 8a–d).

**Figure 9:**
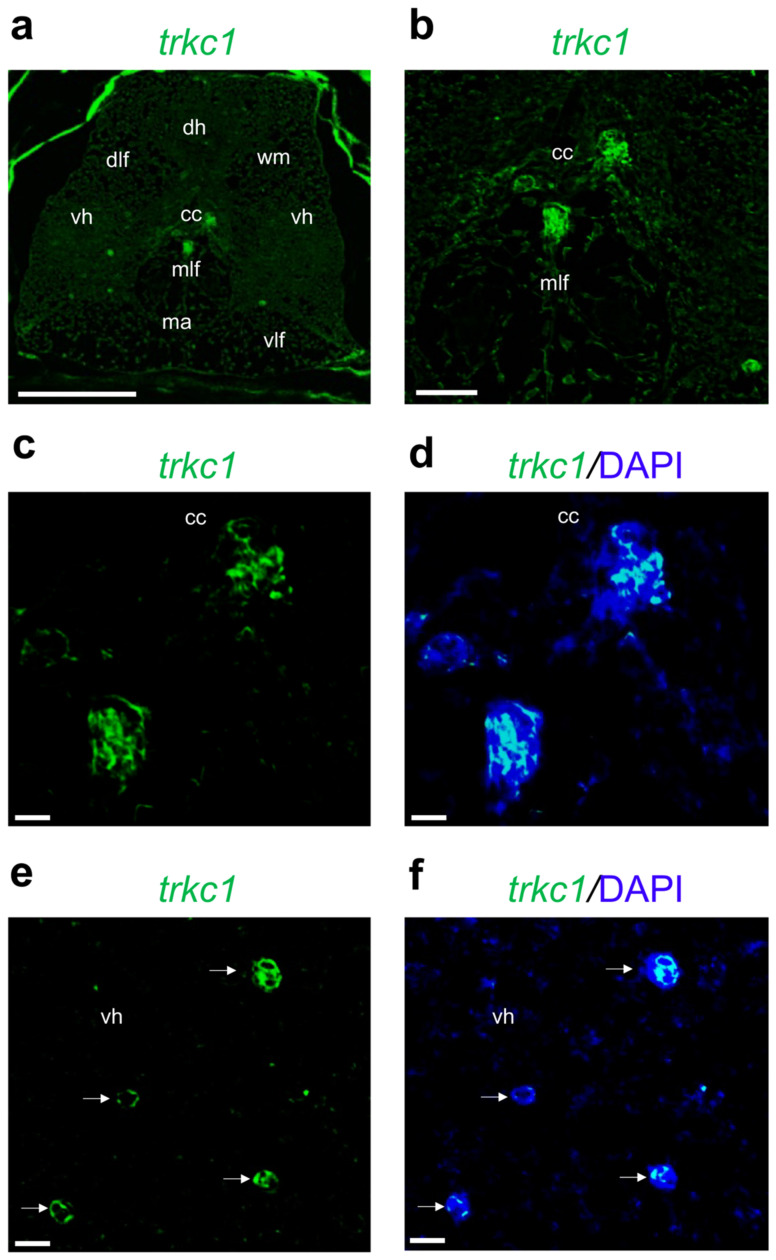
Fluorescence in situ hybridization for *trkc1* in adult zebrafish spinal cord. (**a**) *Trkc1* mRNA distribution in adult zebrafish spinal cord (low magnification of transversal section). (**b**) *Trkc1* mRNA localized in cells very close to the central canal (cc). (**c**) High magnification of *trkc1* mRNA localized in cells very close to the central canal (cc). (**d**) High magnification of *trkc1* mRNA localized in cells very close to the central canal (cc), and cell nuclei are labeled with DAPI (blue). (**e**) High magnification of *trkc1* mRNA localized in cells in ventral horn (vh). (**f**) High magnification of *trkc1* mRNA localized in cells in ventral horn (vh), and cell nuclei are labeled with DAPI (blue). White arrow in (**e**,**f**) indicate *trkc1* expressing cells. Scale bars: 100 μm (**a**); 50 μm (**b**); 25 μm (**c**–**f**).

Finally, *trkc1* mRNA is specifically localized in cells that are very close to the central canal and in a few cells in the ventral horn (Figure 9a–f).

## 3. Discussion

In this study, we described the transcription pattern of neurotrophins and their specific receptors in the adult zebrafish spinal cord. First, we quantified the transcription levels of neurotrophic factors and receptors using qPCR. Our data showed that *ngf* and its receptor *trka* are the most transcribed members in the different regions of the adult zebrafish spinal cord, similarly to the mammalian and chicken spinal cords [44,45]. In zebrafish, we found that *ngf* and its receptor *trka* are more transcribed in cells around the central canal, and in the ventral and dorsal horns. Under normal physiological conditions, in rat and human tissue, *Ngf* and *Trka* mRNA levels are high in the dorsal root ganglia (DRG) as well as in spinal cord [46,47,48]. However, in mice and human patients, after a traumatic spinal cord injury or during the progression of several autoimmune and inflammatory diseases, *Ngf* and *Trka* mRNA levels undergo a dramatic decrease in the spinal cord [49]. Indeed, previous functional studies showed that the *Ngf*/*Trka* signaling pathway can play a crucial role for the regeneration of sensory axons in the adult spinal cord [50]. Concerning the neurotrophic factor *bdnf*, it is more transcribed in the dorsal horn and medial longitudinal fasciculi. This localization pattern is similar to the expression pattern seen in mammals (rat, mouse, and human), as reported in previous studies. Indeed, this neurotrophin is highly expressed in the dorsal horn, where it is involved in the modulation of painful stimuli [51,52]. This result confirms previous studies in other fish, as in the eel, in the rostral spinal cord, large *bdnf*-positive cells were detected throughout the dorsal horn [53]. Interesting, in zebrafish, the *trkb1* receptor is more transcribed in cells that are very close to the central canal and medial longitudinal fasciculi (in this region motor neurons have been identified). This result confirms previous observations in the rat spinal cord. In detail, the authors showed that 20% of the TrkB-positive cell population in the ventral horn resided in close proximity to motor neurons, and were classified as perineuronal [54].

In parallel to other neurotrophins, *nt3* and *nt4/5* in zebrafish were specifically transcribed in two distinct regions. *Nt3* was transcribed in large cells around the central canal. In contrast, *nt4/5* was transcribed starting close to the central canal and sending ipsilateral projections to the ventrolateral edge of the spinal cord. In the mouse, cellular delivery of NT3 promotes corticospinal axonal growth and partial functional recovery after spinal cord injury [55]. Indeed, NT3 can be used to enable chronic-SCI repair and MR-DTI-based mapping of lesion areas [56]. Relative to the role of NT4 in the spinal cord, previous studies showed that, in synergy with BDNF, it could play a crucial role during axon regeneration and motor neuron plasticity [57,58,59]. Finally, we showed the transcription pattern of the *trkc1* receptor, that is transcribed in specific large cells placed near the central canal. Very close to this region, we found *nt3* mRNA-transcribing cells, suggesting that this pathway could be specifically involved in the survival of differentiated cells. Related to the undetected mRNA transcription of neurotrophin *nt6/7* and *trkb2* and *trkc2* receptors in the adult zebrafish spinal cord, this result confirms previous data obtained during zebrafish embryonic development [60], which highlighted very low or undetected mRNA transcriptions of neurotrophin *nt6/7* and *trkb2* and *trkc2* receptors in the spinal cord at the larval stage.

## 4. Materials and Methods

### 4.1. Animals and Spinal Cord Dissection

The present study was conducted on adult males and females (1 year) of *Danio rerio*, zebrafish, obtained from a local furnisher and recently analyzed for other purposes, as described in our recently published study [36]. The animals were housed in a zebrafish aquarium under standard photoperiod conditions (14 h light and 10 h dark) and temperature (28 °C). The zebrafish did not receive medical treatment prior or during the experience. No deaths occurred in the aquarium before the animals were sacrificed for the experiments. All procedures were approved by the Animal Care Committee and authorized by the Italian Ministry of Health (protocol number 2/2020-PR). In detail, the fish were treated with a specific anesthetic (ethyl 3-aminobenzoate and methanesulfonate 0.1%, Sigma Chemicals Co., St. Louis, MO, USA), and the whole spinal cord or segments of it (five in total) were excised.

### 4.2. RNA Extraction

To extract the total RNA, 5 brains and 5 whole spinal cords or segments (5 in total) were pooled and dissociated using RLT buffer with the RNAse minikit (Qiagen, Frankfurt, Germany). To obtain purified RNA, we followed the manufacturer’s protocol. This procedure was repeated in three independent experiments (we used 15 animals in total).

### 4.3. Reverse Transcriptase PCR

For reverse transcription into cDNA, 0.5 μg of total RNA was incubated with a buffer mix and enzyme using the Superscript III First-Strand Synthesis System kit (Invitrogen, Boston, MA, USA). In detail, 10 μL of the total volume was incubated for 10 min at 25 °C, 30 min at 50 °C, and 5 min at 85 °C. Next, the samples were treated with RNAse-H for 30 min at 37 °C.

### 4.4. Quantitative Real-Time PCR

Polymerase chain reaction (PCR) experimental procedures were performed using the thermocycler with the MyiQ detector (Bio-Rad, Hercules, Dallas, TX, USA). We mixed cDNA, specific forward and reverse primers, SYBR-Green (Bio-Rad), and RNase free water according to the manufacturer’s protocol. The previous mix was incubated for 15 min at 95 °C, for 15 s at 95 °C for 40 cycles; for 30 s at 60 °C for 40 cycles; and for 30 s at 72 °C for 40 cycles. For the neurotrophins and receptors we used the following primers as shown in Table 1 and Table 2, respectively.

The data indicate the fold change of transcript levels in the spinal cord (SC) compared with the whole brain (B), using *gapdh* to normalize the absolute quantification, calculated using 2^−∆∆Ct^. To confirm the correct amplification, we performed a melting curve analysis and verified the PCR’s efficiency. Each qPCR experiment was performed using biological triplicates. In the qPCR analyses, each n represents the average of biological triplicates from a single experiment. The experiments were repeated at least three times. No difference in transcription level between females and males was found.

### 4.5. Statistical Analysis

Statistical analysis was completed using the unpaired *t*-test. The software GraphPad Prism version—9.4.1 (458) was used for all statistical analyses. * *p* < 0.01, ** *p* < 0.001, and *** *p* < 0.0001 were considered statistically significant.

### 4.6. Synthesis of Riboprobes for Neurotrophins and Receptors (for In Situ Hybridization)

All digoxigenin (DIG)-labeled antisense riboprobes were generated using the protocol described in our previous studies [36,61] In detail, we produced the following riboprobes: *bdnf*, *ngf*, *nt3*, *nt4/5*, *trka*, *trkb1*, and *trkc1*. No probe was produced for *nt6/7*, *trkb2*, and *trkc2*, for they were undetected by qPCR. The primers that were used to amplify each insert for the neurotrophins and receptors are shown in Table 3 and Table 4, respectively.

After amplification by PCR, each insert was cloned in ZeroBlunt or TOPO-TA vectors (Invitrogen). Next, we performed the transformation into thermocompetent cells. After applying heat shock, the bacteria were plated onto Luria–Bertani (LB) agar plates containing the appropriate antibiotic (respectively ampicillin or kanamycin) to select only the transformed cells. The bacteria transformants were screened, and the white colonies containing the insert were then grown. In detail, the white bacterial colonies were picked and inoculated in LB medium containing the specific antibiotic plates. The bacteria were grown for 16 h in an orbital rotator at 37 °C. Next, we purified DNA plasmid from the contaminations, which were removed using the Quick Plasmid Miniprep Kit (Invitrogen, Boston, MA, USA). We confirmed the antisense and sense orientation using sequencing, and the plasmids were linearized by the appropriate restriction enzymes. We performed in vitro transcription, using T7 polymerase (Roche-Diagnostic) and SP6 polymerase (Roche-Diagnostic, Barrington, IL, USA), with DIG-RNA Labelling Mix (Roche Diagnostic, Indianapolis, IN, USA). Finally, all riboprobes were purified using Nucleo Spin RNA Clean-up columns. We also checked the reaction specificity by hybridizing the sense and antisense riboprobes on adjacent sections.

### 4.7. In Situ Hybridization

The spinal cords were dissected and fixed overnight at 4 °C in 4% PBS/Paraformaldeyde, and processed for paraffin embedding. All of the paraffin sections (7 μm) were deparaffinized using ultraclear, and rehydrated through a series of graded ethanol (100–30%). They were washed in PBS–NaCl (0.85%) and post-fixed for 20 min in PBS–PFA 4%. The spinal cord sections were rinsed in PBS and treated for 10 min at room temperature with proteinase K (2 mg/mL) diluted in PBS. All of the sections were then rinsed as follows: 20 min in 4% PBS-PAF, 10 min in PBS, and 10 min in standard saline citrate (SSC 2x). The sections were incubated overnight at 63 °C in a moist chamber with the probes (1.5 μg/mL) diluted in hybridization buffer, as carried out in previous studies. Finally, the sections were incubated with anti-DIG POD antibody (Roche) at a 1:200 dilution in the above blocking solution overnight at RT. The next day, the sections were washed 4 × 20 min in 1× maleic acid buffer, 4 × 10 min in PBS, and incubated for 1 h in Perkin Elmer amplification diluent buffer. For the reaction, we diluted Tyramide Signal Amplification (TSA plus, Perkin Elmer, Waltham, MA, USA) 1:100 in amplification buffer and Alexa Fluor 488 reagent (Alexa Fluor 488 Tyramide Reagent, Invitrogen) after washing each section three times for 10 min, and then observed them with an epifluorescence microscope (Olympus, equipped with a DP71 digital camera), or a confocal microscope Leica SP2. The images were processed with either the Olympus (Cell) Zeiss (AxioVision4) or Leica (LCS Lite) software V-10.

## Figures and Tables

**Table 1:** Related to Figure 2a.

*bdnf*	F: CGAGGAATAGACAAGCGGCA;	R: ATCCGTATAAACCGCCAGCC
*ngf*	F: GAGAAGACTACAAGCGAAT;	R: CGACAACAATAAGGAGGAT
*nt3*	F: CCCATCAGTGCGCTCATC;	R: TCCGAACTGTCCACCATG
*nt4/5*	F: GCTCCTCCTAGAACAGAG;	R: CGTCCTGGATGCATCTTCT
*nt6/7*	F: GCATTTACAATGGCAGCCAG;	R: CTTCTTGAGTGGTCACTGTC
*gapdh*	F: GCTGGCATCTCCCTCAA	R: TCAGCAACACGATGGCTG

**Table 2:** Related to Figure 2b.

*trka*	F: GCATTTACAATGGCAGCCAG;	R: CTTCTTGAGTGGTCACTGTC
*trkb1*	F: TCACCTATGGCAAGCAACCC	R: CTTTGGGGCAAGTACGAGGT
*trkb2*	F: GAAGTTCTACTCGAATCTCAGG	R: CCAGATGTTCTCACATGCAC
*trkc1*	F: CGGAAGTGGATTGGACAGTT;	R: CATGAAGCCGTTATCGTCC
*trkc2*	F: CTCAAGCATCTTCCAGGGT	R: GATCTGCCGTAGATTGCAG
*gapdh*	F: GCTGGCATCTCCCTCAA	R: TCAGCAACACGATGGCTG

**Table 3:** Related to Figure 3, Figure 4, Figure 5 and Figure 6.

*bdnf*	F: ATAGTAACGAACAGGATGG	R: GCTCAGTCATGGGAGTCC
*ngf*	F: CACAGGAGATCTACGC	R: CGTGGAAAAACCCAACTC
*nt3*	F: TGGTTACCTTTATTACGATC	R: CCACCATTTTTCACGTCC
*nt4/5*	F: CAGAGAAGATGCATCCAGG	R: CGTTTCCTGACACGCG

**Table 4:** Related to Figure 7, Figure 8 and Figure 9.

*trka*	F: ATAGTAACGAACAGGATGG	R: GCTCAGTCATGGGAGTCC
*trkb1*	F: AGAGATGTGTACAGCACC	R: CATTGTTTGAGAGCTGATACC
*trkc1*	F: CACTGAGAGCATCTCTATG	R: CACGTTGTTCATCCCGAC

## Supplementary Materials

The following supporting information can be downloaded at: https://www.mdpi.com/article/10.3390/ijms241310953/s1.

## References

[1] Cohen S., Levi-Montalcini R., Hamburger V. (1954). A Nerve Growth-Stimulating Factor Isolated from Sarcom as 37 and 180. Proc. Natl. Acad. Sci. USA.

[2] Cohen S., Levi-Montalcini R. (1956). A Nerve Growth-Stimulating Factor Isolated from Snake Venom. Proc. Natl. Acad. Sci. USA.

[3] Levi-Montalcini R., Cohen S. (1956). In Vitro and in Vivo Effects of a Nerve Growth-Stimulating Agent Isolated from Snake Venom. Proc. Natl. Acad. Sci. USA.

[4] Ibanez C.F., Hallbook F., Godeau F., Persson H. (1992). Expression of neurotrophin-4 mRNA during oogenesis in Xenopus laevis. Int. J. Dev. Biol..

[5] Berkemeier L.R., Winslow J.W., Kaplan D.R., Nikolics K., Goeddel D.V., Rosenthal A. (1991). Neurotrophin-5: A novel neurotrophic factor that activates trk and trkB. Neuron.

[6] Koliatsos V.E., Cayouette M.H., Berkemeier L.R., Clatterbuck R.E., Price D.L., Rosenthal A. (1994). Neurotrophin 4/5 is a trophic factor for mammalian facial motor neurons. Proc. Natl. Acad. Sci. USA.

[7] Hallbook F. (1999). Evolution of the vertebrate neurotrophin and Trk receptor gene families. Curr. Opin Neurobiol..

[8] Spaink H.P., Jansen H.J., Dirks R.P. (2014). Advances in genomics of bony fish. Brief. Funct. Genom..

[9] Hallbook F., Lundin L.G., Kullander K. (1998). Lampetra fluviatilis neurotrophin homolog, descendant of a neurotrophin ancestor, discloses the early molecular evolution of neurotrophins in the vertebrate subphylum. J. Neurosci..

[10] Tettamanti G., Cattaneo A.G., Gornati R., de Eguileor M., Bernardini G., Binelli G. (2010). Phylogenesis of brain-derived neurotrophic factor (BDNF) in vertebrates. Gene.

[11] De Girolamo P., D’Angelo L. (2021). Neurotrophins in the Brain of Teleost Fish: The State of the Art. Adv. Exp. Med. Biol..

[12] Dos Santos S., Mazan S., Venkatesh B., Cohen-Tannoudji J., Querat B. (2011). Emergence and evolution of the glycoprotein hormone and neurotrophin gene families in vertebrates. BMC Evol. Biol..

[13] Nilsson A.S., Fainzilber M., Falck P., Ibanez C.F. (1998). Neurotrophin-7: A novel member of the neurotrophin family from the zebrafish. FEBS Lett..

[14] Jaillon O., Aury J.M., Brunet F., Petit J.L., Stange-Thomann N., Mauceli E., Bouneau L., Fischer C., Ozouf-Costaz C., Bernot A. (2004). Genome duplication in the teleost fish Tetraodon nigroviridis reveals the early vertebrate proto-karyotype. Nature.

[15] Martin S.C., Sandell J.H., Heinrich G. (1998). Zebrafish TrkC1 and TrkC2 receptors define two different cell populations in the nervous system during the period of axonogenesis. Dev. Biol..

[16] Martin S.C., Marazzi G., Sandell J.H., Heinrich G. (1995). Five Trk receptors in the zebrafish. Dev. Biol..

[17] Cacialli P. (2021). Neurotrophins Time Point Intervention after Traumatic Brain Injury: From Zebrafish to Human. Int. J. Mol. Sci..

[18] Scarisbrick I.A., Isackson P.J., Windebank A.J. (1999). Differential expression of brain-derived neurotrophic factor, neurotrophin-3, and neurotrophin-4/5 in the adult rat spinal cord: Regulation by the glutamate receptor agonist kainic acid. J. Neurosci..

[19] Skup M., Dwornik A., Macias M., Sulejczak D., Wiater M., Czarkowska-Bauch J. (2002). Long-term locomotor training up-regulates TrkB(FL) receptor-like proteins, brain-derived neurotrophic factor, and neurotrophin 4 with different topographies of expression in oligodendroglia and neurons in the spinal cord. Exp. Neurol..

[20] Rahman M.M., Islam M.R., Supti F.A., Dhar P.S., Shohag S., Ferdous J., Shuvo S.K., Akter A., Hossain M.S., Sharma R. (2023). Exploring the Therapeutic Effect of Neurotrophins and Neuropeptides in Neurodegenerative Diseases: At a Glance. Mol. Neurobiol..

[21] Ricci S., Cacialli P. (2021). Stem Cell Research Tools in Human Metabolic Disorders: An Overview. Cells.

[22] Guo W., Zhang X., Zhai J., Xue J. (2022). The roles and applications of neural stem cells in spinal cord injury repair. Front. Bioeng. Biotechnol..

[23] Choi T.Y., Choi T.I., Lee Y.R., Choe S.K., Kim C.H. (2021). Zebrafish as an animal model for biomedical research. Exp. Mol. Med..

[24] Cacialli P., Lucini C. (2019). Adult neurogenesis and regeneration in zebrafish brain: Are the neurotrophins involved in?. Neural Regen. Res..

[25] El-Daher F., Becker C.G. (2020). Neural circuit reorganisation after spinal cord injury in zebrafish. Curr. Opin Genet. Dev..

[26] Hui S.P., Nag T.C., Ghosh S. (2020). Neural cells and their progenitors in regenerating zebrafish spinal cord. Int. J. Dev. Biol..

[27] Tsata V., Wehner D. (2021). Know How to Regrow-Axon Regeneration in the Zebrafish Spinal Cord. Cells.

[28] Tsata V., Kroehne V., Wehner D., Rost F., Lange C., Hoppe C., Kurth T., Reinhardt S., Petzold A., Dahl A. (2020). Reactive oligodendrocyte progenitor cells (re-)myelinate the regenerating zebrafish spinal cord. Development.

[29] Shen W.Y., Fu X.H., Cai J., Li W.C., Fan B.Y., Pang Y.L., Zhao C.X., Abula M., Kong X.H., Yao X. (2022). Identification of key genes involved in recovery from spinal cord injury in adult zebrafish. Neural Regen. Res..

[30] Menke A.L., Spitsbergen J.M., Wolterbeek A.P., Woutersen R.A. (2011). Normal anatomy and histology of the adult zebrafish. Toxicol. Pathol..

[31] Zeng C.W., Sheu J.C., Tsai H.J. (2020). The Neuronal Regeneration of Adult Zebrafish after Spinal Cord Injury Is Enhanced by Transplanting Optimized Number of Neural Progenitor Cells. Cell Transplant..

[32] Aragona M., Porcino C., Guerrera M.C., Montalbano G., Laura R., Cometa M., Levanti M., Abbate F., Cobo T., Capitelli G. (2022). The BDNF/TrkB Neurotrophin System in the Sensory Organs of Zebrafish. Int. J. Mol. Sci..

[33] Aragona M., Porcino C., Guerrera M.C., Montalbano G., Levanti M., Abbate F., Laura R., Germana A. (2021). Localization of Neurotrophin Specific Trk Receptors in Mechanosensory Systems of Killifish (*Nothobranchius guentheri*). Int. J. Mol. Sci..

[34] Cacialli P., Gueguen M.M., Coumailleau P., D’Angelo L., Kah O., Lucini C., Pellegrini E. (2016). BDNF Expression in Larval and Adult Zebrafish Brain: Distribution and Cell Identification. PLoS ONE.

[35] Cacialli P., D’Angelo L., de Girolamo P., Avallone L., Lucini C., Pellegrini E., Castaldo L. (2018). Morpho-Functional Features of the Gonads of Danio rerio: The Role of Brain-Derived Neurotrophic Factor. Anat. Rec..

[36] Cacialli P. (2022). Expression of Nerve Growth Factor and Its Receptor TrkA in the Reproductive System of Adult Zebrafish. Vet. Sci..

[37] Cacialli P., Gatta C., D’Angelo L., Leggieri A., Palladino A., de Girolamo P., Pellegrini E., Lucini C. (2019). Nerve growth factor is expressed and stored in central neurons of adult zebrafish. J. Anat..

[38] Gatta C., Schiano V., Attanasio C., Lucini C., Palladino A. (2022). Neurotrophins in Zebrafish Taste Buds. Animals.

[39] Germana A., Gonzalez-Martinez T., Catania S., Laura R., Cobo J., Ciriaco E., Vega J.A. (2004). Neurotrophin receptors in taste buds of adult zebrafish (Danio rerio). Neurosci. Lett..

[40] Cacialli P., Lucini C. (2022). Analysis of the Expression of Neurotrophins and Their Receptors in Adult Zebrafish Kidney. Vet. Sci..

[41] Gatta C., Altamura G., Avallone L., Castaldo L., Corteggio A., D’Angelo L., de Girolamo P., Lucini C. (2016). Neurotrophins and their Trk-receptors in the cerebellum of zebrafish. J. Morphol..

[42] Mollmert S., Kharlamova M.A., Hoche T., Taubenberger A.V., Abuhattum S., Kuscha V., Kurth T., Brand M., Guck J. (2020). Zebrafish Spinal Cord Repair Is Accompanied by Transient Tissue Stiffening. Biophys. J..

[43] Stil A., Drapeau P. (2016). Neuronal labeling patterns in the spinal cord of adult transgenic Zebrafish. Dev. Neurobiol..

[44] Large T.H., Weskamp G., Helder J.C., Radeke M.J., Misko T.P., Shooter E.M., Reichardt L.F. (1989). Structure and developmental expression of the nerve growth factor receptor in the chicken central nervous system. Neuron.

[45] Ip F.C., Cheung J., Ip N.Y. (2001). The expression profiles of neurotrophins and their receptors in rat and chicken tissues during development. Neurosci. Lett..

[46] Averill S., McMahon S.B., Clary D.O., Reichardt L.F., Priestley J.V. (1995). Immunocytochemical localization of trkA receptors in chemically identified subgroups of adult rat sensory neurons. Eur. J. Neurosci..

[47] Mu X., Silos-Santiago I., Carroll S.L., Snider W.D. (1993). Neurotrophin receptor genes are expressed in distinct patterns in developing dorsal root ganglia. J. Neurosci..

[48] Josephson A., Widenfalk J., Trifunovski A., Widmer H.R., Olson L., Spenger C. (2001). GDNF and NGF family members and receptors in human fetal and adult spinal cord and dorsal root ganglia. J. Comp. Neurol..

[49] Funakoshi H., Frisen J., Barbany G., Timmusk T., Zachrisson O., Verge V.M., Persson H. (1993). Differential expression of mRNAs for neurotrophins and their receptors after axotomy of the sciatic nerve. J. Cell Biol..

[50] Hajebrahimi Z., Mowla S.J., Movahedin M., Tavallaei M. (2008). Gene expression alterations of neurotrophins, their receptors and prohormone convertases in a rat model of spinal cord contusion. Neurosci. Lett..

[51] Ramer M.S., Priestley J.V., McMahon S.B. (2000). Functional regeneration of sensory axons into the adult spinal cord. Nature.

[52] Lever I.J., Bradbury E.J., Cunningham J.R., Adelson D.W., Jones M.G., McMahon S.B., Marvizon J.C., Malcangio M. (2001). Brain-derived neurotrophic factor is released in the dorsal horn by distinctive patterns of afferent fiber stimulation. J. Neurosci..

[53] Eriksson N.P., Aldskogius H., Grant G., Lindsay R.M., Rivero-Melian C. (1997). Effects of nerve growth factor, brain-derived neurotrophic factor and neurotrophin-3 on the laminar distribution of transganglionically fransported choleragenoid in the spinal cord dorsal horn following transection of the sciatic nerve in the adult rat. Neuroscience.

[54] Dalton V.S., Roberts B.L., Borich S.M. (2009). Brain derived neurotrophic factor and trk B mRNA expression in the brain of a brain stem-spinal cord regenerating model, the European eel, after spinal cord injury. Neurosci. Lett..

[55] Coulibaly A.P., Deer M.R., Isaacson L.G. (2014). Distribution and phenotype of TrkB oligodendrocyte lineage cells in the adult rat spinal cord. Brain Res..

[56] Bao S.S., Zhao C., Chen H.W., Feng T., Guo X.J., Xu M., Rao J.S. (2022). NT3 treatment alters spinal cord injury-induced changes in the gray matter volume of rhesus monkey cortex. Sci. Rep..

[57] Zhao C., Rao J.S., Duan H., Hao P., Shang J., Fan Y., Zhao W., Gao Y., Yang Z., Sun Y.E. (2022). Chronic spinal cord injury repair by NT3-chitosan only occurs after clearance of the lesion scar. Signal Transduct. Target Ther..

[58] Keefe K.M., Sheikh I.S., Smith G.M. (2017). Targeting Neurotrophins to Specific Populations of Neurons: NGF, BDNF, and NT-3 and Their Relevance for Treatment of Spinal Cord Injury. Int. J. Mol. Sci..

[59] Sakuma K., Watanabe K., Sano M., Uramoto I., Nakano H., Li Y.J., Kaneda S., Sorimachi Y., Yoshimoto K., Yasuhara M. (2001). A possible role for BDNF, NT-4 and TrkB in the spinal cord and muscle of rat subjected to mechanical overload, bupivacaine injection and axotomy. Brain Res..

[60] Long S.L., Liu F., Wang T.H., Xu X.Y., Guang Y.G., Wang T.W., Ke Q., Yuan Y. (2005). The changes of NT-4 expression in spared root ganglion and spinal cord following partial dorsal root rhizotomy. Sichuan Da Xue Xue Bao Yi Xue Ban.

[61] Nittoli V., Sepe R.M., Coppola U., D’Agostino Y., De Felice E., Palladino A., Vassalli Q.A., Locascio A., Ristoratore F., Spagnuolo A. (2018). A comprehensive analysis of neurotrophins and neurotrophin tyrosine kinase receptors expression during development of zebrafish. J. Comp. Neurol..

