# Supplementary material for: Transcription Pattern of Neurotrophic Factors and Their Receptors in Adult Zebrafish Spinal Cord
Source: Int J Mol Sci. 2023 Jun 30;24(13):10953. doi: 10.3390/ijms241310953 (PMC10341495; doi:10.3390/ijms241310953)
Supplement: Supplementary file 1 [file ijms-24-10953-s001.zip › ijms-2469957-supplementary.pdf]

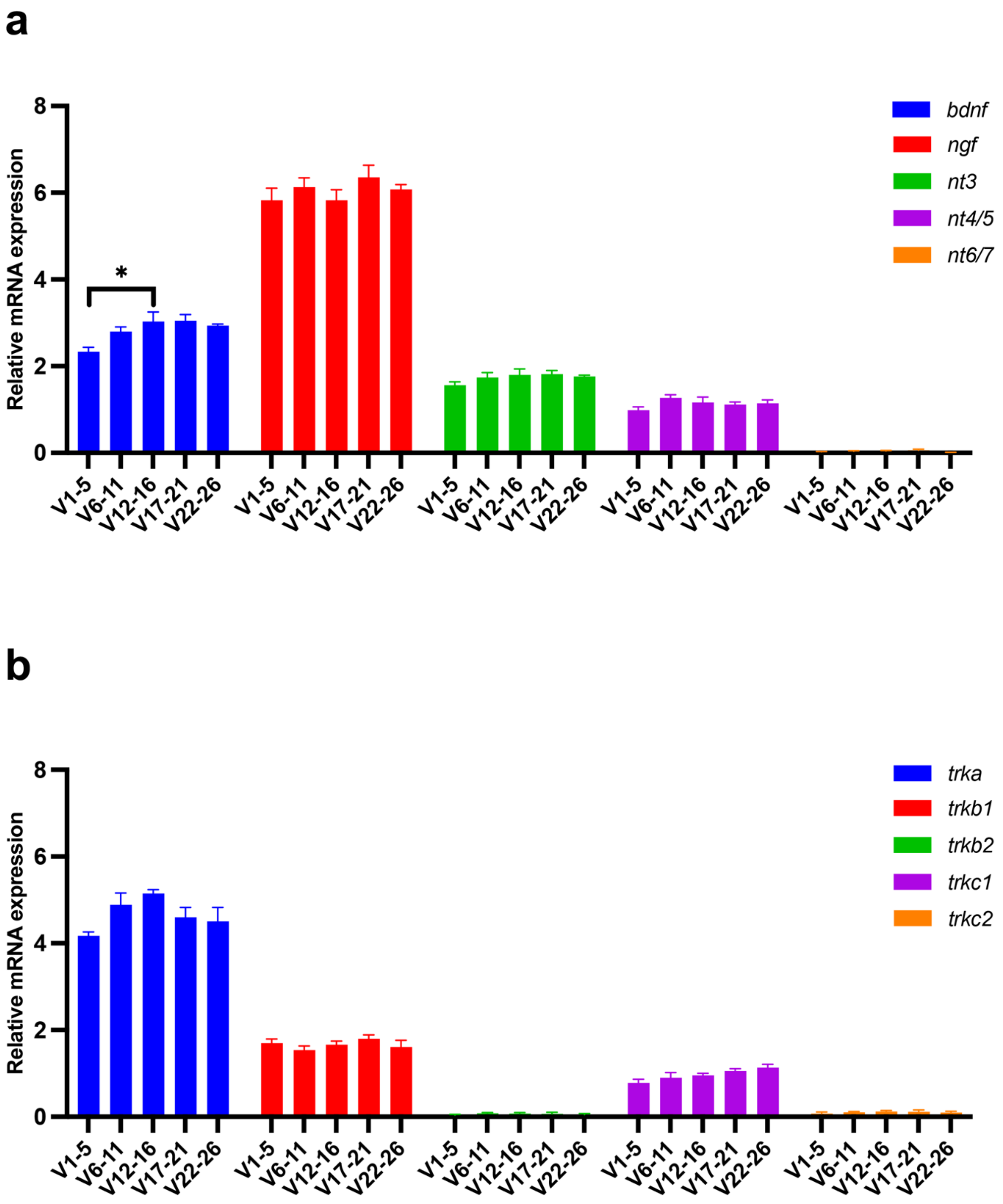

Figure S1. qPCR analysis of neurotrophins and receptors in five different segments of adult zebrafish spinal cord. (a) *Ngf* is highly transcribed in all regions of spinal cord. *Bdnf* transcription level presents a slight variation (the level is lower in V1-5 compared to the other segments) *P < 0.01. *Nt3* does not show variation in transcription level between different regions. It is the same for *nt4*. *Nt6/7* is undetectable in all regions. Statistical analysis was completed using a one-way ANOVA, adjusted for multiple test comparison (n=5 animals used). (b) The receptor *trka* is equally transcribed in all regions of spinal cord. Similarly, *trkb1* does not present significative difference in transcription level between the different segments. *Trkc1* presents a low level of transcription in all regions of spinal cord. *Trkb2* and *trkc2* are undetectable in all segments. Statistical analysis was completed using a one-way ANOVA, adjusted for multiple test comparison (n=5 animals used).
